# Distribution patterns of influenza virus receptors and viral attachment patterns in the respiratory and intestinal tracts of seven avian species

**DOI:** 10.1186/1297-9716-43-28

**Published:** 2012-04-10

**Authors:** Taiana Costa, Aida J Chaves, Rosa Valle, Ayub Darji, Debby van Riel, Thijs Kuiken, Natàlia Majó, Antonio Ramis

**Affiliations:** 1 Departament de Sanitat i Anatomia Animals, Facultat de Veterinària, Universitat Autònoma de Barcelona, Bellaterra, Barcelona, Spain; 2 Centre de Recerca en Sanitat Animal, UAB-IRTA, Campus de la Universitat Autònoma de Barcelona, Bellaterra, Barcelona, Spain; 3 Institut de Recerca i Tecnologia Agroalimentàries, Barcelona, Spain; 4 Department of Virology, Erasmus Medical Center, Rotterdam, The Netherlands

## Abstract

This study assessed the presence of sialic acid α-2,3 and α-2,6 linked glycan receptors in seven avian species. The respiratory and intestinal tracts of the chicken, common quail, red-legged partridge, turkey, golden pheasant, ostrich, and mallard were tested by means of lectin histochemistry, using the lectins *Maackia amurensis *agglutinin II and *Sambucus nigra *agglutinin, which show affinity for α-2,3 and α-2,6 receptors, respectively. Additionally, the pattern of virus attachment (PVA) was evaluated with virus histochemistry, using an avian-origin H4N5 virus and a human-origin seasonal H1N1 virus. There was a great variation of receptor distribution among the tissues and avian species studied. Both α-2,3 and α-2,6 receptors were present in the respiratory and intestinal tracts of the chicken, common quail, red-legged partridge, turkey, and golden pheasant. In ostriches, the expression of the receptor was basically restricted to α-2,3 in both the respiratory and intestinal tracts and in mallards the α-2,6 receptors were absent from the intestinal tract. The results obtained with the lectin histochemistry were, in general, in agreement with the PVA. The differential expression and distribution of α-2,3 and α-2,6 receptors among various avian species might reflect a potentially decisive factor in the emergence of new viral strains.

## Introduction

Wild aquatic birds are generally considered to be the source of all influenza viruses found in mammal and avian species, including humans, pigs, horses, minks, marine mammals, cats, and a great number of domestic avian species [1]. Phylogenetic studies indicate that all human influenza viruses, including the predominant strains associated with the seasonal flu (H1, H2, and H3 subtypes) originated from an avian ancestor [2]. More recently, a swine-origin influenza A H1N1 virus (pH1N1), which contains genes of human, avian and swine influenza viruses, caused the first pandemic of the 21^st ^century [3].

The host restriction of influenza A viruses is in part determined by specific sialic acid receptors on the surface of susceptible cells. These receptors are composed of nine carbon monosaccharides, usually found on the outermost terminal position of glycan chains, linked to cell-surface glycoproteins and glycolipids [4]. The N-acetylneuraminic acid (Neu5Ac), one of the most common sialic acids, is usually bound to galactose (Gal) in an α-2,3 (Neu5ACα-2,3Gal) or α-2,6 configuration (Neu5ACα-2,6Gal), and their expression and distribution are cell specific [5]. The affinity of influenza viruses for these receptors varies according to the species from which they are isolated. Influenza viruses of avian origin preferentially bind to Neu5Acα-2,3Gal (α-2,3 receptors, avian-like receptors), the form that predominates in the duck enteric tract where these viruses replicate [6,7]; whereas human influenza strains recognize Neu5ACα-2,6Gal (α-2,6 receptors, human-like receptors) [8,9].

For many years, it was thought that the inter-species barrier could only be crossed after adaptation of an avian influenza virus in pigs, since pigs were shown to harbor both α-2,3 and α-2,6 receptors [6,10]. Later on, it was observed that the H5 and H7 avian influenza virus subtypes could be directly transmitted from poultry to humans, in spite of having α-2,3 receptor specificity [11-13]. This observation encouraged investigators to study the role of α-2,3 and α-2,6 receptors in the species barrier, and led to the description of α-2,3 receptors in the human lower respiratory tract, which may partially explain the localization and severity of H5N1-associated pneumonia in humans [14,15].

The expression of influenza receptors in avian and mammal species have been studied by means of lectin histochemistry, based on the binding affinity of *Maackia amurensis *agglutinin II (MAAII) for α-2,3 receptors [16], and of the plant-derived lectin *Sambucus nigra *agglutinin (SNA), which preferentially detects α-2,6 receptors [17]. The avian species that have been studied include the following: the chicken (*Gallus gallus domesticus*), common quail (*Coturnix coturnix*), Japanese quail (*Coturnix japonica*), bobwhite quail (*Colinus virginianus*), Chinese ring-necked pheasants (*Phasianus colchicus*), turkey (*Meleagris gallopavo*), pearl guinea fowl, Pekin duck (*Anas platythynchos domestica*), mallard (*Anas platyrhynchos*), Tolousse goose (*Anser anser domesticus*), black-headed gull (*Larus ridibundus*), mew gull (*Larus canus*), herring gull (*Larus argentatus*), domestic pigeon (*Columba livia*), common wood pigeon (*Columba palumbus*), dunlin (*Calidris alpina*), and common murre (*Uria aalge*) [7,18-26]. The presence of both the α-2,3 and α-2,6 receptors has been reported in some domestic avian species such as the chicken, bobwhite quail, turkey, Chinese ring neck pheasant, white midget turkey, Pearl guinea fowl, and Pekin duck [18,19,23,25,27]. However, the information available in these studies is generally restricted to a few tissues in the respiratory or intestinal tracts, and accompanied by a concise description of the pattern expression of influenza receptors.

The expression of influenza virus receptors can also be evaluated with virus histochemistry to determine the pattern of viral attachment (PVA) [9]. Virus histochemistry is a binding assay based on the attachment of concentrated fluorescein-labeled virus on host cells, and visualized by routine immunohistochemical techniques. The PVA is a valuable tool to determine the affinity of a specific virus to certain species and host cells, which are critical factors for an effective infection [15]. Several recent studies have evaluated the PVA of influenza viruses in avian species [28], as well as in humans [15,29-31] and other mammal species [29]. However, current knowledge of the PVA in avian species is still very limited [28].

The combined use of both techniques, lectin histochemistry and virus histochemistry, could be useful to elucidate the role of domestic species in the transmission of influenza viruses and help to understand the evolutionary pressures exerted by different poultry species over influenza viruses, discerning for instance, why these viruses evolve faster in chickens and turkeys than in wild birds [32]. Furthermore, understanding viral and host barriers that prevent transmission may be critical in establishing rational control measures as well as predicting and stratifying risk for individual strains of influenza [33].

The present study extensively assessed the expression of α-2,3 and α-2,6 receptors in the respiratory (nasal cavity, trachea, and lung) and intestinal (duodenum, jejunum-ileum, cecum, and colon) tracts of seven domestic avian species. Furthermore, the PVA of an avian-origin H4N5 virus and a human-origin H1N1 influenza virus on respiratory and intestinal tracts was also evaluated. The species studied include the chicken, common quail, red-legged partridge (*Alectoris rufa*), turkey, golden pheasant (*Chrysolophus pictus*), ostrich (*Struthio camelus*), and mallard. These domestic species were selected because they are commonly held commercially for egg, meat, feather or leather production, or for ornamental purposes. The influence of the α-2,3 and α-2,6 receptor-distribution and PVA on the emergence and perpetuation of influenza viruses is discussed herein.

## Materials and methods

### Animals and tissues

The seven domestic avian species included in this study were the following: chicken, common quail, red-legged partridge, turkey, golden pheasant, ostrich, and mallard. Three individuals of each species were used. Samples of the nasal cavity (including both middle and posterior turbinates), trachea, lung, duodenum, jejunum, ileum, cecum and colon were obtained from archival formalin-fixed paraffin-embedded tissues of the Veterinary Pathology Service of the *Universitat Autònoma de Barcelona *(Barcelona, Spain). Tissues free from any histopathological lesions were selected for this study. Human, pig and mice tissue samples were used as positive controls for the detection of α-2,3 and α-2,6 receptors. Human lung samples, obtained from an adult patient that died without previous pulmonary disease, were kindly provided by *Hospital Universitari Vall d'Hebrón *(Barcelona, Spain) in accordance with protocols approved by the Ethics Committee on Clinic Investigations of the Hospital. Respiratory and intestinal tracts of pigs and mice were obtained from animals that died without previous respiratory or digestive diseases, submitted for necropsy at the Veterinary Pathology Service of the *Universitat Autònoma de Barcelona*. This study was carried out in strict accordance with the recommendations of the Ethics Committee of Animal and Human Experimentation of the *Universitat Autònoma de Barcelona*.

### Lectin histochemistry

Lectin histochemistry was performed as previously described [34] with minor modifications. Briefly, 3 μm-thick sections were deparaffinized and treated with 3% H_2_O_2 _in methanol to eliminate endogenous peroxidase activity, washed with Tris-NaCl-Tween buffer (TNT) (0.1 M Tris HCl, 0.15 M NaCl, pH 7.5), and blocked with TNB (TNT plus blocking reagent) (Perkin Elmer, US) for 30 min at room temperature (RT). Tissue sections were then incubated with biotinylated SNA (10 μg/mL) and MAAII (15 μg/mL) (Vector Laboratories Inc, CA, USA) in TNB at 4 °C, overnight. After washing with TNT, sections were incubated with streptavidin-horse radish peroxidase (SA-HRP) 1:100 for 1 h, followed by incubation with Tyramide Signal Amplification (TSA™ Biotin System, Perkin Helmer, USA) at 1:50 in dilution Buffer, and again incubated with SA-HRP for 30 min at RT. The reaction was developed with diaminobenzidine (Sigma-Aldrich, MO, USA) at RT for 30 s followed by counterstaining with Mayer's haematoxylin. The expression of the receptors was visible by light microscopy as brown staining. To rule out the non-specific binding of lectins, two sequential slides were used as negative controls. One slide was pretreated with neuraminidase (NA), which cleaves both α-2,3 and α-2,6 residues, as previously described [34]; and the other was incubated with phosphate buffered saline instead of the lectins. After careful examination of each slide, and in order to compare receptor expression patterns among the tissues and species included in this study, the relative intensity of receptor expression was scored based on the percentage of cells in a section showing positivity, and was graded as: negative (-); low (+), when 1% or more, but less than 10% of the cells were positive; moderate (++), when 10% or more, but less than 50% of the cells were positive; strong (+++), when 50% or more of the cells were positive. Photomicrography of the lectin histochemistry was taken using a Leica DM6000B microscope, Leica DFC480 digital camera, and Leica Application Suite software program.

### Virus histochemistry

The attachment of influenza virus to epithelial cells in respiratory and digestive tracts was visualized by virus histochemistry as previously described [29]. With the exception of the chicken and mallard, where three individuals were included, one individual of each species was evaluated using the virus histochemistry technique. The viruses used were A/Mallard/Netherlands/13/08 (H4N5) and the seasonal A/Netherlands/35/05 (H1N1). Both viruses were prepared as previously described [29]. Briefly, the viruses were inoculated in chicken embryo chorioallantoic membrane (H4N5) or in Madin-Darby canine kidney cells (H1N1), and harvested two days later. Viruses were concentrated and purified by centrifugation on sucrose gradient, inactivated by dialysis against 0.1% formalin, and labeled with fluorescein isothiocyanate (FITC). Tissue sections were incubated with 50-100 hemagglutinating units per 50 μL of FITC-labeled virus. Attachment of virus was detected with a peroxidase-labeled anti-FITC antibody, and amplified with a tyramide signal amplification system. Peroxidase was revealed with 3-amino-9-ethyl-carbazole, which resulted in a bright red precipitate. Attachment of influenza viruses to tissues was visible by light microscopy as granular to diffuse red staining on the apical surface and in the cytoplasm of epithelial cells. As for the lectin histochemistry, the relative intensity of the viral attachment to epithelial cells was scored based on the percentage of cells in a section showing virus attachment, as follows: negative (-); low (+), when 1% or more, but less than 10% of the cells were positive; moderate (++), when 10% or more, but less than 50% of the cells were positive; strong (+++), when 50% or more of the cells were positive. Photomicrography of the virus histochemistry was taken using a Leica DM6000B microscope, Leica DFC480 digital camera, and Leica Application Suite software program.

## Results

### Lectin histochemistry

The pattern of receptor expression in the control tissues used in this study (human lung, pig and mice respiratory and digestive tracts) was in agreement with previously published literature. The NA pretreatment, used as a control, removed all the binding sites for the SNA; however, very low levels of staining for MAAII remained after the NA treatment in connective tissue of the lamina propria in the respiratory and intestinal tracts of all the species studied, and was considered as non-specific staining (data not shown).

#### Respiratory tract

**Figure 1:**
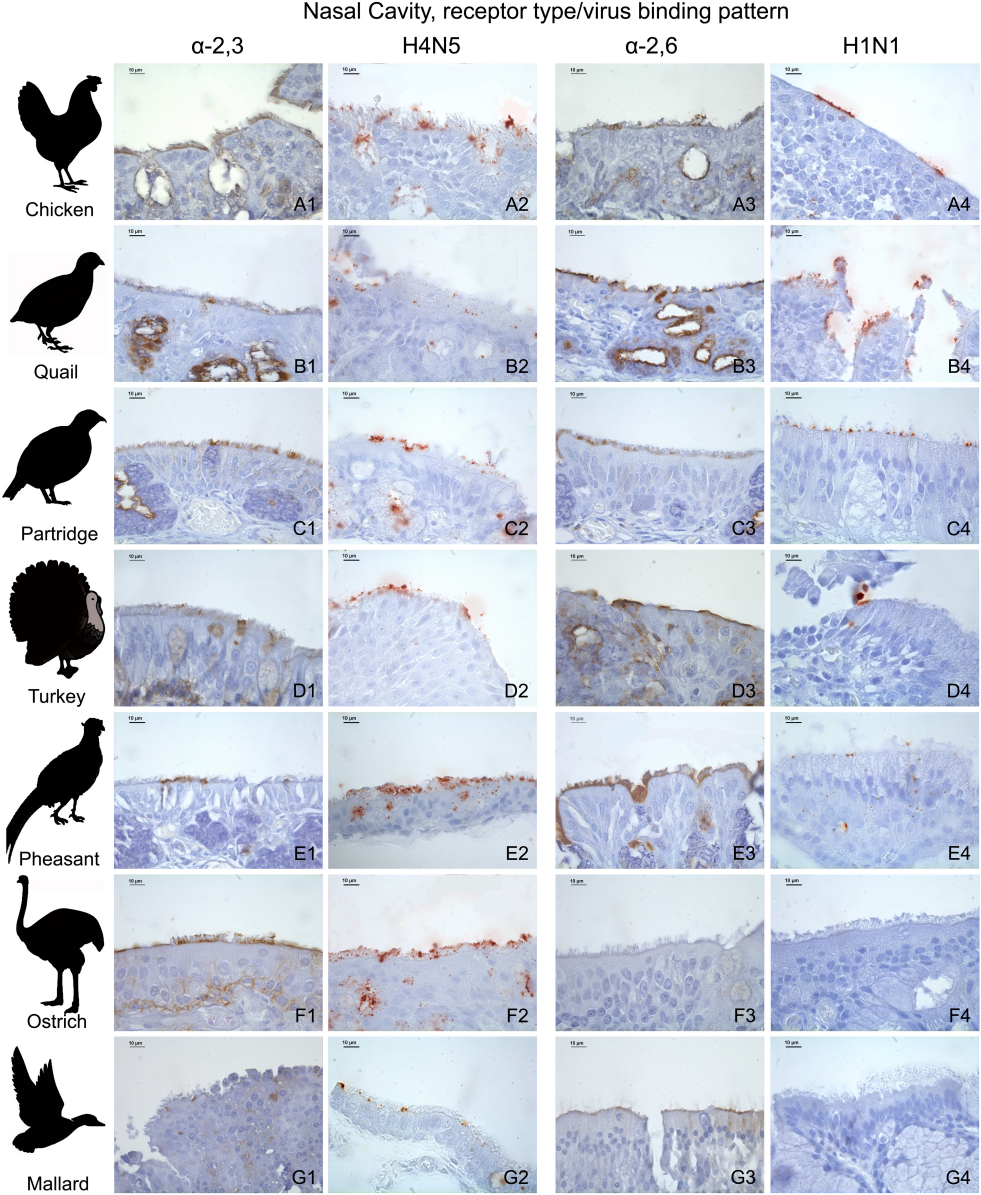
**Influenza receptor distribution and pattern of viral attachment in the nasal cavity**. Composite bright field microscope images comparing the distribution of α-2,3 and α-2,6 receptors, demonstrated by means of MAAII and SNA lectin histochemistry, with the pattern of viral attachment of the avian influenza A/Mallard/Netherlands/13/08 (H4N5) virus and the human influenza A/Netherlands/35/05 (H1N1) virus, demonstrated by means of virus histochemistry, in the nasal cavity of the chicken (A1-A4), common quail (B1-B4), red-legged partridge (C1-C4), turkey (D1-D4), golden pheasant (E1-E4), ostrich (F1-F4), and mallard (G1-G4).

**Figure 2:**
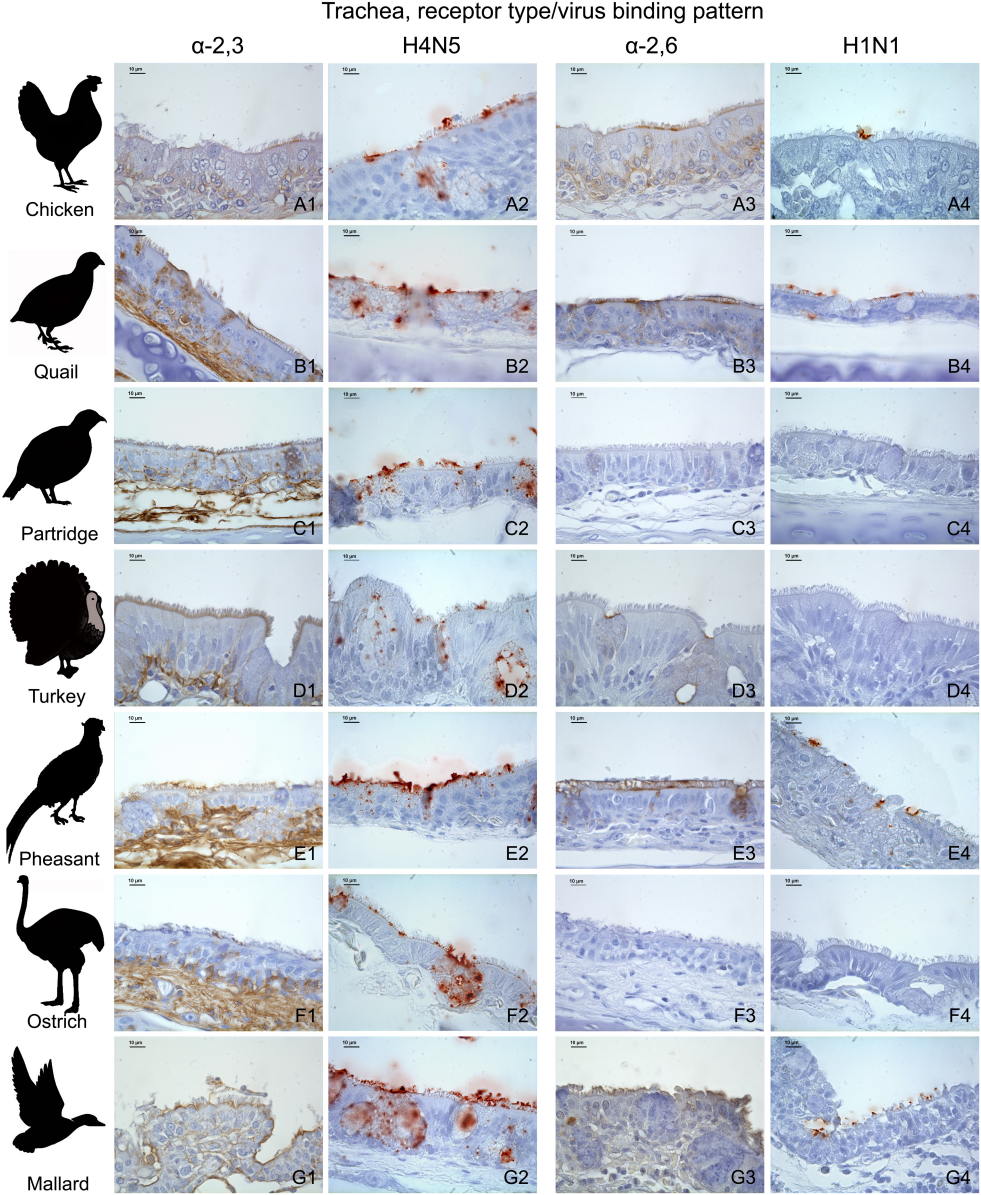
**Influenza receptor distribution and pattern of viral attachment in the trachea**. Composite bright field microscope images comparing the distribution of α-2,3 and α-2,6 receptors, demonstrated by means of MAAII and SNA lectin histochemistry, with the pattern of viral attachment of the avian influenza A/Mallard/Netherlands/13/08 (H4N5) virus and the human influenza A/Netherlands/35/05 (H1N1) virus, demonstrated by means of virus histochemistry, in the trachea of chicken (A1-A4), common quail (B1-B4), red-legged partridge (C1-C4), turkey (D1-D4), golden pheasant (E1-E4), ostrich (F1-F4), and mallard (G1-G4).

There was a marked variation on the distribution and expression of influenza receptors among the tissues and avian species studied. The distribution of influenza receptors in the respiratory tract is given in Table 1 and the results from nasal cavity and trachea are illustrated in Figures 1 and 2, respectively.

**Table 1:** Distribution of Sia α-2,3 Gal and Sia α-2,6 Gal receptors^a ^in the upper and lower respiratory tracts of seven avian species.

Tissue, cell type	Species, receptor type^b^													
	
	Chicken		Common Quail		Red-legged Partridge		Turkey		Golden Pheasant		Ostrich		Mallard	
	
	α-2,3	α-2,6	α-2,3	α-2,6	α-2,3	α-2,6	α-2,3	α-2,6	α-2,3	α-2,6	α-2,3	α-2,6	α-2,3	α-2,6
**Nasal Cavity**														

*Respiratory Epithelium*														

Ciliated epithelial cells	+++	++	+	+++	++	+	++	++	++	++	++	-	+	+

Non-ciliated epithelial cells	+	-	+	++	+	+	-	+	+	+	+	-	++	-

*Olfactory Epithelium*														

Olfactory epithelial cells	+++	+	+	++	++	++	++	++	+	++	++	-	-	-

Bowman gland epithelium	+++	+	++	++	++	+	-	+	+	-	-	-	++	+

*Adjacent structures*														

Nasal gland epithelium	+	++	++	+	+	+	++	++	++	+	nd	nd	++	+

Salivary gland epithelium	++	+	+++	++	+	+	++	++	++	+	+++	-	++	+

**Trachea**														

Ciliated epithelial cells	+	+	+	+++	+	-	+++	-	-	++	+	-	++	++

Goblet cells	-	+	+	+	+	-	-	+	+	+	+	-	-	++

Mucous gland epithelium	++	+	+	+	+	+	+	+	+	+	+	-	-	-

**Lung**														

Bronchial epithelial cells	+++	+++	+	+++	-	-	++	+++	+	+	+	-	+	+

Parabronchial epithelial cells	+++	-	+	-	-	-	-	+++	+	-	+	-	+	-

Air capillary cells	-	-	+	-	-	+	-	-	-	+	+	-	-	-

^a ^The distribution of α-2,3 and α-2,6 influenza receptors was evaluated using lectin immunohistochemistry.

^b ^-: negative; +: low; ++: moderate; +++: strong; nd: not determined.

##### Chicken

In the nasal cavity, strong α-2,3 receptors were observed on respiratory ciliated epithelial cells as well as on olfactory epithelial cells, but were low on respiratory non-ciliated epithelial cells. In contrast, positivity for α-2,6 receptors was moderate on respiratory ciliated epithelial cells and low on olfactory epithelial cells. Chicken trachea showed low levels of staining for both receptors on ciliated epithelial cells. The chicken lung manifested strong staining for both receptors in the bronchial epithelial cells, whereas the parabronchial epithelial cells were strongly positive for α-2,3 receptors only.

##### Common quail

In common quails, low levels of α-2,3 receptors were observed in the nasal cavity (respiratory epithelium and olfactory epithelial cells), trachea (ciliated epithelial cells) and lung (bronchial and parabronchial epithelial cells), whereas strong expression of α-2,6 receptors was observed in nasal, tracheal and bronchial epithelial cells. Expression of α-2,6 receptors on respiratory non-ciliated epithelial cells and olfactory epithelium was relatively moderate.

##### Red-legged partridge

The partridge respiratory tract showed moderate staining for α-2,3 receptors on respiratory ciliated epithelial cells and the olfactory epithelium, low staining on respiratory non-ciliated epithelial cells and tracheal ciliated epithelial cells, and negative staining in the lung. Likewise, expression of α-2,6 receptors was moderate on olfactory epithelial cells, low on the respiratory epithelium, and negative on tracheal and pulmonary epithelial cells.

##### Turkey

The turkey nasal cavity expressed moderate staining for both receptors on respiratory ciliated epithelial cells and olfactory epithelial cells, while low levels of staining for α-2,6 receptors was observed on non-ciliated epithelial cells. Tracheal ciliated epithelial cells showed strong expression of α-2,3 receptors and negative expression for α-2,6 receptors. Moderate positivity for α-2,3 receptors was observed on bronchial epithelial cells, while strong positivity for α-2,6 receptors was observed in bronchial and parabronchial epithelial cells.

##### Golden pheasant

Moderate α-2,3 receptor expression was observed on respiratory ciliated epithelial cells while low levels of α-2,3 receptor expression were observed on respiratory non-ciliated epithelial cells, olfactory epithelium and lung (bronchial and parabronchial epithelial cells). Similarly, moderate positivity for α-2,6 receptors was observed on respiratory ciliated epithelial cells, olfactory epithelial cells, and tracheal ciliated epithelial cells; while low levels of staining were observed on respiratory non-ciliated epithelial cells and bronchial epithelial cells.

##### Ostrich

Low levels of α-2,3 receptor expression were observed in the trachea (ciliated epithelial cells), lung (bronchial and parabronchial epithelial cells), as well as respiratory non-ciliated epithelial cells. Moderate expression of α-2,3 receptors was noticed on respiratory ciliated epithelial cells and olfactory epithelial cells. Interestingly, α-2,6 receptors were not expressed in any segment of the ostrich respiratory tract. The receptor expression was not determined on the ostrich nasal gland epithelium due to the lack of this structure on the samples evaluated.

##### Mallard

Moderate expression of α-2,3 receptors was observed in respiratory non-ciliated epithelial cells and tracheal ciliated epithelial cells; while low levels of expression of α-2,3 receptors was noted on nasal respiratory ciliated epithelial cells, and bronchial and parabronchial epithelial cells. Moderate expression of α-2,6 receptors was observed in tracheal ciliated epithelial cells and low levels of α-2,6 receptor expression were observed in respiratory ciliated epithelial cells and bronchial epithelial cells.

#### Intestinal tract

**Figure 3:**
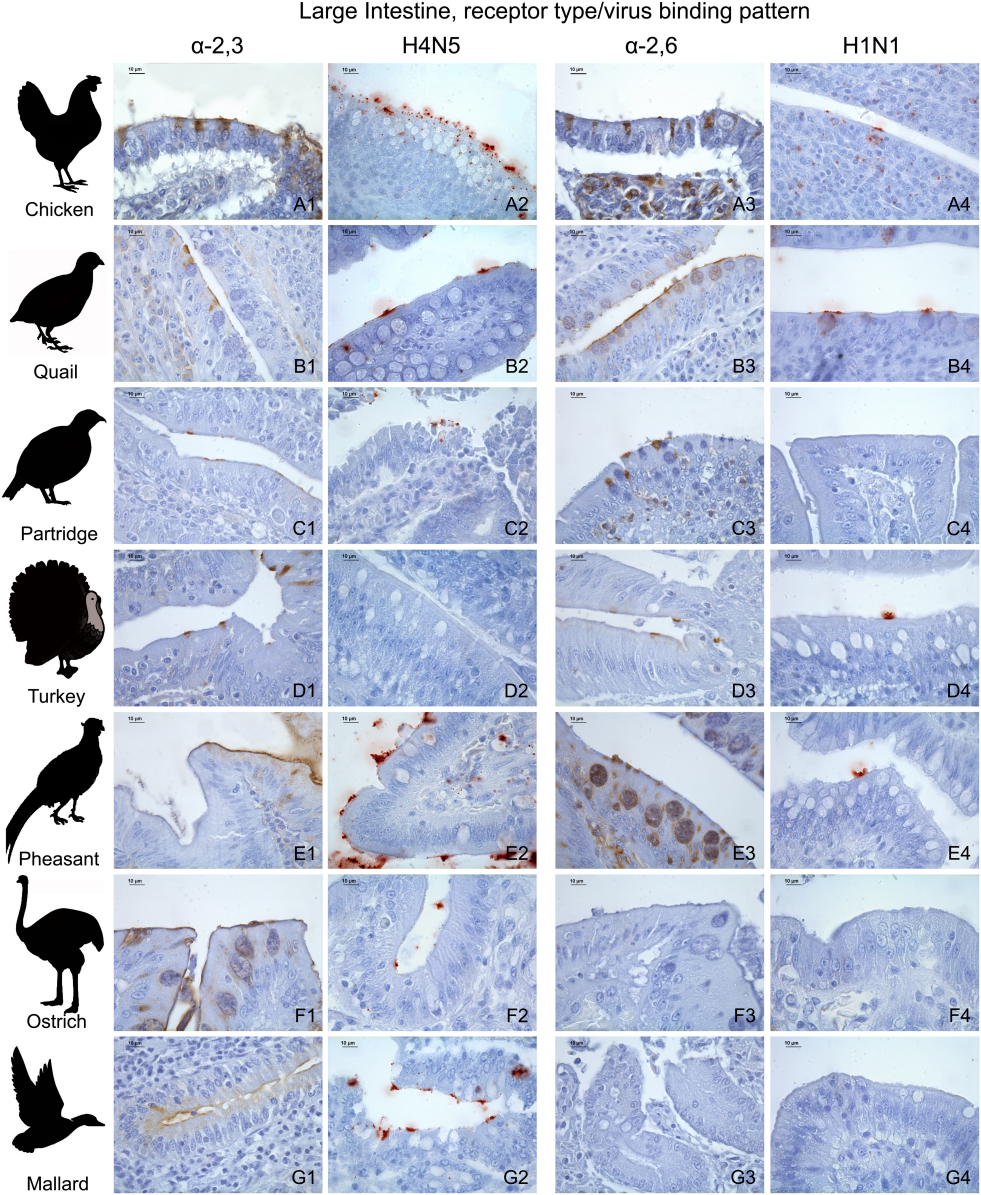
**Influenza receptor distribution and pattern of viral attachment in the large intestine**. Composite bright field microscope images comparing the distribution of α-2,3 and α-2,6 receptors, demonstrated by means of MAAII and SNA lectin histochemistry, with the pattern of viral attachment of the avian influenza A/Mallard/Netherlands/13/08 (H4N5) virus and the human influenza A/Netherlands/35/05 (H1N1) virus, demonstrated by means of virus histochemistry, in the large intestine of the chicken (A1-A4), common quail (B1-B4), red-legged partridge (C1-C4), turkey (D1-D4), golden pheasant (E1-E4), ostrich (F1-F4), and mallard (G1-G4).

The expression and distribution of influenza receptors in the intestinal tract is described in detail in Table 2 and the results from the large intestine are illustrated in Figure 3.

**Table 2:** Distribution of Sia α-2,3 Gal and Sia α-2,6 Gal receptors^a ^in the intestinal tract of seven avian species.

Tissues, cell type	Species, receptor type^b^													
	
	Chicken		Common Quail		Red-legged Partridge		Turkey		Golden Pheasant		Ostrich		Mallard	
	
	α-2,3	α-2,6	α-2,3	α-2,6	α-2,3	α-2,6	α-2,3	α-2,6	α-2,3	α-2,6	α-2,3	α-2,6	α-2,3	α-2,6
**Duodenum**														

Columnar epithelial cells	++	-	+	+	-	-	+	-	++	+	+	-	+++	-

Goblet cells	+++	-	++	+	-	+	-	-	+	+	++	-	-	-

GALT lymphocytes	-	+	-	+	+	+	+	+	+	+	-	+	+	-

**Jejunum-Ileum**														

Columnar epithelial cells	++	+	+	+	+	-	+	-	++	+	+	-	+++	-

Goblet cells	+++	-	++	-	-	-	+	-	+	+	++	-	-	-

**Cecum**														

Columnar epithelial cells	+	+	++	++	+	+	-	+	++	+	+	-	+++	-

Goblet cells	-	-	-	+	-	-	-	-	+	++	++	-	-	-

GALT lymphocytes	-	++	-	+	-	+	-	+	-	+	-	+	-	-

**Colon**														

Columnar epithelial cells	+	-	++	+	+	-	+	+	++	+	-	-	+++	-

Goblet cells	-	-	-	+	-	+	-	-	-	++	++	-	-	-

GALT lymphocytes	-	++	-	-	-	-	+	-	+	+	-	+	-	-

^a ^The distribution of α-2,3 and α-2,6 influenza receptors was done using lectin immunohistochemistry.

^b ^-: negative; +: low; ++: moderate; +++: strong.

##### Chicken

Moderate positivity for α-2,3 receptors was observed on columnar epithelial cells of the small intestine (duodenum and jejunum-ileum), and low levels of staining were recorded on columnar epithelial cells from the large intestine (cecum and colon). Expression of α-2,6 receptors was low on columnar epithelial cells of the jejunum-ileum and cecum.

##### Common quail

Moderate expression of α-2,3 receptors was observed on columnar epithelial cells from the large intestine, while low levels of expression of α-2,3 receptors were observed in columnar epithelial cells from the small intestine. Regarding the expression of α-2,6 receptors, a moderate staining was visualized in cecal columnar epithelial cells, and low levels of staining were present in columnar epithelial cells from the small intestine and colon.

##### Red-legged partridge

Both receptors were expressed in low levels in red-legged partridge intestinal tract. The partridge intestinal tract showed expression of α-2,3 receptors along the columnar epithelial cell from the jejunum-ileum, cecum and colon, while α-2,6 receptors were only observed in cecal columnar epithelial cells.

##### Turkey

Both receptors were expressed in low levels in turkey intestinal tract. Expression of α-2,3 receptors was noted on columnar epithelial cells from the small intestine and colon, while expression of α-2,6 was restricted to columnar epithelial cells from the large intestine.

##### Golden pheasant

Moderate expression of α-2,3 receptors was observed in columnar epithelial cells from the small and large intestines, while expression of α-2,6 receptors was low on columnar epithelial cells from the small and large intestines.

##### Ostrich

The α-2,3 receptors were predominant on the intestinal tract. The expression of α-2,3 receptors was low on columnar epithelium cells from the small intestine and cecum, while α-2,6 receptors were absent from intestinal epithelial cells.

##### Mallard

Strong expression of α-2,3 receptors was observed throughout the columnar epithelial cells from the small and large intestines. The α-2,6 receptors were not expressed in the intestinal tract of mallards.

### Virus histochemistry

The expression of α-2,3 and α-2,6 receptors, as determined by lectin histochemistry, was compared with the PVA of avian-origin H4N5 and human-origin H1N1 influenza viruses in the respiratory tract (Table 3) and intestinal tract (Table 4) of the seven avian species used in this study. In order to facilitate the comparison between the lectin histochemistry and virus histochemistry, the lectin histochemistry results in these tables were summarized; a media of the scoring of the epithelia lining (ciliated and non-ciliated epithelial cells) and goblet cells of the different tissues from the respiratory and intestinal tracts was calculated and expressed as follows: nasal cavity (nasal turbinates and infraorbital sinuses), trachea, lung, small intestine, and large intestine. It is remarkable that the H1N1 virus did not attach to the respiratory tract of the ostrich, nor did the H4N5 virus on the intestinal tract of turkeys. The results obtained with the receptor expression and the PVA were in general comparatively consistent among the different tissues and avian species studied (Tables 3 and 4, Figures 1, 2 and 3). With the exception of several cases, the avian-origin A/Mallard/Netherlands/13/08 (H4N5) virus bound to tissues where α-2,3 receptors were observed, and the human-origin A/Netherlands/35/05 (H1N1) virus bound to tissues where α-2,6 receptors were observed. In the respiratory tract of all the species evaluated, the attachment of A/Mallard/Netherlands/13/08 (H4N5) was more evident than the attachment of A/Netherlands/35/05 (H1N1), particularly in the nasal cavity and trachea (Table 3 Figures 1 and 2). However, this tendency was not observed for the intestinal tract (Table 4 Figure 3).

**Table 3:** Influenza receptor distribution^a ^and pattern of viral attachment^b ^in the respiratory tract^c ^of different avian species.

	Tissues, types of receptor/virus binding^d^											
**Species**	**Nasal Cavity^e^**				**Trachea**				**Lung**			

	α**-2,3**	**H4N5**	α**-2,6**	**H1N1**	α**-2,3**	**H4N5**	α**-2,6**	**H1N1**	α**-2,3**	**H4N5**	α**-2,6**	**H1N1**

**Chicken**	++	++^f^	+	++	+	++	+	+	+++	+	++	+

**Quail**	+	+	++	++	++	++	++	+	+	++^g^	+	+

**Partridge**	++	++	+	+	+	++	-	-	-	+	+	-

**Turkey**	+	++	++	+	++	++	+	-	+	+	+++	+

**Pheasant**	+	+++	++	+	+	+++	++	+	+	+	+	-

**Ostrich**	++	+++	-	-	+	++	-	-	+	+	-	-

**Mallard**	+	+	+	-	+	+++	++	+	+	++ ^g^	+	-

^a ^The distribution of α-2,3 and α-2,6 influenza receptors was done using lectin immunohistochemistry.

^b ^The pattern of viral attachment was performed using virus histochemistry and the A/Mallard/Netherlands/13/08 (H4N5) and A/Netherlands/35/05 (H1N1) viruses.

^c ^Media of the results obtained in the epithelia lining (ciliated and non-ciliated epithelial cells and goblet cells).

^d ^-: negative; +: low; ++: moderate; +++: strong.

^e ^'Nasal Cavity' includes the following: nasal turbinates and infraorbital sinuses.

^f ^Virus binding mainly in infraorbital sinuses.

^g ^Virus binding mainly in bronchi.

**Table 4:** Influenza receptor distribution^a ^and pattern of viral attachment^b ^in the intestinal tract^c ^of different avian species.

Species	Tissues, types of receptor/virus binding^d^							
	
	Small Intestine				Large Intestine			
	
	α-2,3	H4N5	α-2,6	H1N1	α-2,3	H4N5	α-2,6	H1N1
**Chicken**	+++	++	+	+	+	++	+	+

**Quail**	++	+	+	+	+	+	++	+

**Partridge**	+	+	+	+	+	+	+	-

**Turkey**	+	-	-	+	+	-	+	+

**Pheasant**	++	+	+	+	++	+++	++	+

**Ostrich**	++	+	-	+	++	+	-	-

**Mallard**	++	+	-	+	++	+	-	-

^a ^The distribution of α-2,3 and α-2,6 influenza receptors was evaluated using lectin immunohistochemistry.

^b ^The pattern of viral attachment was performed using virus histochemistry and the A/Mallard/Netherlands/13/08 (H4N5) and A/Netherlands/35/05 (H1N1) viruses.

^c ^Media of the results obtained in the epithelia lining (columnar epithelial cells and goblet cells).

^d ^-: negative; +: low; ++: moderate; +++: strong.

Minor inconsistencies between lectin histochemistry and PVA results were detected, and consisted in one grade of scoring. In some cases, there was viral attachment despite the absence of receptor expression, as observed in the partridge lung for α-2,3/H4N5 (Table 3) and in turkey, ostrich, and mallard small intestine for α-2,6/H1N1 (Table 4); in all these cases, the tissue was negative for the presence of the receptor but showed a PVA graded as low. Conversely, in other cases there was no viral attachment despite the presence of receptor expression, as that occurring for α-2,6/H1N1 in the mallard nasal cavity, turkey trachea, and partridge, pheasant and mallard lungs (Table 3); as well as turkey small and large intestines for α-2,3/H4N5, and partridge large intestine for α-2,6/H1N1 (Table 4). In these cases, the tissue showed low expression of the receptor, but was negative for the PVA.

## Discussion

In this study, a lectin histochemistry technique was used to extensively assess the expression of α-2,3 and α-2,6 receptors in the respiratory and intestinal tracts of seven domestic avian species. The staining for these two types of receptors was not performed in a single assay and, therefore, it is not possible to accurately determine whether cells co-express both α-2,3 and α-2,6 receptors, but to infer about the expression of these receptors in a particular tissue or cell type. In the chicken, common quail, red-legged partridge, turkey, and golden pheasant, both α-2,3 and α-2,6 receptors were expressed in at least one segment of the respiratory and intestinal tracts. The α-2,6 receptors were not observed in the respiratory tract of the ostrich, nor in the intestinal tract of mallards. The PVA of an avian-origin H4N5 and a human-origin H1N1 influenza virus was also evaluated on the same tissues and compared with the lectin histochemistry distribution pattern. There was a great variation among the tissues and avian species studied (Tables 1 and 2), and, with a few exceptions, the results obtained with the lectin histochemistry were in agreement with the virus histochemistry (Tables 3 and 4).

Virus histochemistry has shown to be a useful assay to study the pattern of virus attachment in different tissues, and the PVA and the lectin histochemistry results were comparatively consistent. When both techniques were compared, the results obtained with the PVA and with the lectin histochemistry were either equal or varied only one grade (Tables 3 and 4). There were only two cases where the lectin histochemistry was graded as strong (+++) and the PVA was graded as low (+), and only three cases where the lectin histochemistry was graded as low (+) and the PVA was graded as strong (+++) (Table 3).

The results obtained in the chicken were in agreement with previous reports, in which a limited number of tissues were evaluated [21,24,27]. However in the present study we observed a low level of staining for the α-2,6 receptor on the epithelial cell in the jejunum-ileum and cecum, whereas Liu et al. [20] did not detect α-2,6 receptors in the intestinal tract of chickens, and Kuchipudi et al. [21] only detected this receptor in the large intestine. These differences could be attributed to the signal amplification methodology used in our study. On the contrary, the presence of α-2,6 receptors and the binding of the human-origin H1N1 virus to the intestinal tract of chickens was in accordance with the results of an experimental study where a human-origin influenza A virus was able to bind in vitro to chicken colon cells [27].

In common quails, our results were in accordance with previous reports in common quails [26] and Japanese quails [23,25] in which both α-2,3 and α-2,6 receptors were observed in the respiratory and intestinal tracts. Another study with bobwhite quail, however, did not detect an α-2,6 receptor in the intestinal tract [19]. This difference in receptor expression could be related to interspecies differences. Both viruses used for the PVA were able to bind to the respiratory and intestinal tracts of quails, particularly the H4N5 virus in trachea.

In red-legged partridges, low levels of expression of both α-2,3 and α-2,6 receptors were observed in respiratory and intestinal tracts. Assuming this pattern of receptor distribution is seen in other partridge species, it could explain the susceptibility of chukar partridges (*Alectoris chukar*) to experimental infection with both avian-origin and swine-origin influenza viruses [35].

In turkeys, both α-2,3 and α-2,6 receptors were detected on epithelial cells along the intestinal tract and the entire respiratory tract, in agreement with recent studies [18,19,36]. Regarding the PVA, attachment of the human-origin H1N1 virus was observed in the respiratory (nasal cavity and lung) and intestinal tracts, while the attachment of the avian-origin H4N5 virus was restricted to the respiratory tract. This was consistent with the fact that turkeys are also susceptible to swine-origin H1N1 and H3N2 influenza viruses [37-41], to reassortant viruses with human, swine, and avian influenza genes (H1N2) [42,43], as well as to the pH1N1 virus, as demonstrated in August 2009 in two turkey flocks naturally infected in Chile [44], indicating the potential of avian species that express both α-2,3 and α-2,6 receptors, to be susceptible to mammal-origin influenza viruses and potentially offer an adequate environment for the emergence of reassortant viruses. Turkeys have also been successfully infected with the pH1N1 virus by experimental inoculation via the intrauterine route, with subsequent oropharyngeal and cloacal virus shedding, but were not infected when the intranasal route was used [45]. This observation shows that other factors rather than the tissue distribution of receptors and the affinity of virus binding may determine the outcome of an exposure to certain influenza A viruses.

In golden pheasants, the observed expression of both α-2,3 and α-2,6 receptors in the respiratory and intestinal tracts was in accordance with previous reports [19,26]. This may explain why pheasants can be infected with avian-origin influenza viruses that have specificity to α-2,6 receptors, as observed for some H9N2 influenza virus isolates [46]. In pheasants, the PVA of the avian-origin H4N5 was observed throughout the respiratory and intestinal tract, while the PVA of the human-origin H1N1 virus was restricted to the trachea and intestinal tract. In addition, the fact that ring-necked pheasants could not be experimentally infected with human and swine influenza viruses [35] indicates that, as mentioned above, besides the hemagglutinin receptor binding site, other factors may be involved in the restriction of inter-species transmission [47].

In ostriches, the influenza receptor expression was almost exclusively restricted to α-2,3 receptors on epithelial cells throughout the respiratory and intestinal tracts, as with the PVA of the avian-origin H4N5 virus. The expression of α-2,6 receptors was low on GALT lymphocytes, and the PVA of the human-origin H1N1 virus was restricted to a few lining epithelial cells of the small intestine. In fact, there are no reports of ostrich infection with mammal-origin influenza viruses.

In mallards, both α-2,3 and α-2,6 receptors were observed in the respiratory tract, as previously reported [19,22,26]. Regarding the intestinal tract, α-2,3 receptors were moderately expressed in mallards, while α-2,6 receptors were absent. A recent study, however, reported a very minimal expression of α-2,6 receptors in the large intestine of the Pekin duck [19]. The absence of abundant α-2,6 receptors in the mallard intestinal tract, associated with the fact that the PVA of the human-origin H1N1 virus was low and restricted to the trachea and small intestine, correlate with the fact that ducks are resistant to the infection with human influenza A viruses under natural and experimental conditions [5,48].

When interpreting the distribution of influenza receptors based on lectin histochemistry or virus histochemistry it is important to take several factors into consideration. For instance, studies of influenza virus infections in *ex vivo *cultures indicated that there are alternative receptors that cannot be identified with the lectins used in the present study [49]. Furthermore, ongoing viral mutations and adaptation may change host receptor affinity over time, further limiting lectin staining [50]. Additionally, besides the distribution of receptors, other host and viral factors intervene in the process of viral replication and adaptation of any influenza viruses in a new species, such as host immune response, and viral glycoproteins and internal proteins [47,51].

Using glycan micro arrays it has been shown that not all α-2,3 or α-2,6 receptors bind to influenza hemagglutinin (HA) proteins equally well; one glycan terminating in α-2,3 might not bind HA while another may bind exceedingly well [52]. Therefore, determining the influenza virus-binding profile in tissues of different animal species is a condition fundamental to better understanding the role of these receptors [19]. However, care must be taken when interpreting and extrapolating PVA results, since the PVA may vary between and within influenza A strains.

In summary, this study demonstrates that although both α-2,3 and α-2,6 receptors are expressed in domestic birds, there is marked variation among species. This information helps to understand the effect of host pressure on virus evolution and indicates that "mixing vessels" is not only restricted to pigs, since other species could also have an important role in the transmission and adaptation of influenza viruses of avian-origin to the mammal host. Hence these poultry species could pose a greater threat to humans, since avian viruses with affinity for α-2,6 receptors may be selected, amplified and transmitted. This could explain why some avian H9N2 strains acquired affinity for α-2,6 receptors in quail after continual circulation in the field [46,53,54]. In this respect, it is important to determine the role of concomitantly co-expressed α-2,3 and α-2,6 receptors in the emergence of new viral strains, especially those with pandemic potential.

## Abbreviations

FITC: Fluorescein isothiocyanate; Gal: Galactose; GALT: Gut-associated lymphoid tissue; FITC: Fluorescein isothiocyanate; GALT: Gut-associated lymphoid tissue; HA: Hemagglutinin; IHC: Immunohistochemistry; MAAII: *Maackia amurensis *agglutinin II; NA: Neuraminidase; Neu5Ac: N-acetylneuraminic acid; PVA: Pattern of viral attachment; RT: Room temperature; SA-HRP: Streptavidin-horse radish peroxidase; SNA: *Sambucus nigra *agglutinin; TNT: Tris-NaCl-Tween buffer.

## Competing interests

The authors declare that they have no competing interests.

## Authors' contributions

TC, AJC, NM and AR prepared the manuscript. AJC and RV performed the lectin histochemistry assay. AJC and TC carried out the lectin histochemistry evaluation. AR, TK and DVR performed the virus histochemistry. TC and AR carried out the virus histochemistry evaluation. AJC, AD, NM and AR conceived the study and participated in its design and coordination. All authors read and approved the final manuscript.
